# Chicken liver and eggshell crackers as a safe and affordable animal source food for overcoming micronutrient deficits during pregnancy and lactation in Indonesia: a double-blind, randomised placebo-controlled trial (SISTIK Growth Study)

**DOI:** 10.12688/wellcomeopenres.17879.1

**Published:** 2022-06-09

**Authors:** Aly Diana, Sofa Rahmannia, Yenni Zuhairini Suhadi, Dimas Erlangga Luftimas, Haidar Rizqi, Afini Dwi Purnamasari, Ayunda Jihadillah, Mohammad Brachim Ansari, Dearly Ayu Zahrotun Haq, Aisyah Nur Pratiwi, Samuel Scott, Daniela Hampel, Lindsay H Allen, Jillian J Haszard, Lisa A Houghton, Rosalind S Gibson, Umi Fahmida

**Affiliations:** 1 Department of Public Health, Faculty of Medicine, Universitas Padjadjaran, Bandung, 40161, Indonesia; 2 Nutrition Working Group, Faculty of Medicine, Universitas Padjadjaran, Bandung, 40161, Indonesia; 3 Southeast Asian Ministers of Education Organization Regional Center for Food and Nutrition (SEAMEO RECFON), Pusat Kajian Gizi Regional Universitas Indonesia, Jakarta, 13120, Indonesia; 4 Faculty of Medicine, Universitas Pasundan, Bandung, 40117, Indonesia; 5 School of Population and Global Health, University of Western Australia, Crawley, Western Australia, 6009, Australia; 6 Poverty Health and Nutrition Division, International Food Policy Research Institute, Poverty Health and Nutrition Division, New Delhi, 110012, India; 7 Department of Nutrition, University of California, One Shields Ave, Davis, 95616, USA; 8 USDA, ARS-Western Human Nutrition Research Center, Davis, CA, 95616, USA; 9 Biostatistics Centre, University of Otago, Dunedin, 9054, New Zealand; 10 Department of Human Nutrition, University of Otago, Dunedin, 9016, New Zealand

**Keywords:** food based intervention, Indonesia, lactation, linear growth, micronutrient deficiencies, pregnancy, stunting

## Abstract

**Background:** Indonesia ranks fifth in terms of the number of stunted children and there has been little change in the stunting prevalence in the last decade. In earlier observational studies conducted in 2014-2015, we identified several key underlying problems with the potential to impact stunting in Sumedang district, West Java, Indonesia. Deficits in intakes of growth-limiting micronutrients were observed, most notably calcium, iron, zinc, and vitamin A, emphasizing the need for a food-based intervention to overcome these micronutrient deficits in the diets of mothers and their infants.

**Methods:** A double-blind placebo-controlled cluster randomised trial comparing the effect of daily consumption of 75 grams of locally produced micronutrient-enriched crackers (MEC) (intervention group) compared to placebo crackers (control group) by mothers at two-time intervals: (i) from the 8-14 weeks of pregnancy to delivery (i.e., 28-34 weeks of consumption of MEC) on birth length, and (ii) from the 8-14 weeks of pregnancy to 5 months post-partum on attained linear growth and linear growth velocity of breast-fed infants. A total of 324 pregnant women from 28 clusters (villages) located in 3 sub-districts in Sumedang district, West Java, Indonesia, will be randomly assigned to either intervention (n=14 villages) or control (n=14 villages).

**Discussi** **on:** This will be the first study in Indonesia to use crackers based on powdered eggshells and chicken liver, in a form which is acceptable, safe, and has a long shelf life. If daily consumption of MEC for 6 months during pregnancy can enhance birth length, or their continued daily consumption for 5 months postpartum improves both attained and incremental linear growth at 5 months of age, then scaling-up in Indonesia may be considered.

**Trial Registration**: https://clinicaltrials.gov/ct2/show/NCT04564222 ; 25 ^th^ September 2020

## Introduction

Reduction in child stunting is the first goal of the WHO Global Nutrition Targets for 2025 and a key indicator in Sustainable Development Goal # 2. Indonesia is a country with the fifth highest burden of stunted children in the world ^1^, yet there has been a negligible change in the stunting prevalence in the last decade. Furthermore, large disparities exist in the prevalence of stunting across provinces in Indonesia, emphasising the urgent need to investigate the aetiology of linear growth retardation sub-nationally, so that tailored intervention strategies can be implemented effectively. Linear growth retardation and stunting are associated with (but do not cause) delays in cognitive and motor development in childhood, reduced earnings in adulthood, and chronic diseases ^2^.

In earlier observational studies conducted in 2014–2015, we identified several key underlying problems with the potential to impact stunting in Sumedang district, West Java, Indonesia. In Sumedang, the prevalence of stunting in children less than five years of age was 32.2% in 2018, comparable to the national average (30.8%) at that time. Deficits in intakes of growth-limiting micronutrients were observed, most notably calcium, iron, zinc, and vitamin A ^3^, some of which were associated with anaemia (15% and 39%) and co-existing micronutrient deficiencies such as zinc (60% and 10%), iron (25% and 67%), and vitamin A (34% and 13%) deficiency in both lactating mothers ^4, 5^ and their infants ^6^, respectively. We identified several factors likely to contribute to these micronutrient inadequacies and with the potential to exacerbate the risk of stunting during infancy. These factors included low intakes of animal-source foods among both mothers and infants ^7, 8^, sub-optimal concentrations of some breastmilk micronutrient concentrations ^4, 5^, and risk of infant exposure to faecal contamination from unsafe household drinking water ^9^. For example, in our longitudinal study in Sumedang of breastfed infants, length-for-age Z-scores at 12 mos were positively associated with consumption of iron-rich/fortified infant foods ^7^, but negatively associated with “so-called” household access to improved drinking water, which we later showed was a source of faecal contamination ^9^. Moreover, there was a high prevalence of elevated faecal myeloperoxidase (MPO) in infant stool samples collected at 12 months. Faecal MPO is a marker for intestinal inflammation and its associated increases in intestinal permeability and reductions in nutrient absorption, a condition called environmental enteric dysfunction (EED). These disturbances arise from a damaged gut due to repeated exposure to pathogens and appear to be associated with the risk of micronutrient deficiencies that are independent of diet and systemic inflammation ^10^. Hence, it is conceivable that EED also had a role in the decline in LAZ of these resource-poor Sumedang infants.

Food-based intervention is needed to overcome the micronutrient deficits in the diets of mothers and their infants. Iron and folic acid supplementation have become part of the public health program. However, the compliance has been low (~50%) ^11^. Therefore, we have developed and pretested the acceptability of micronutrient-enriched crackers (MEC); they have a long shelf life and are now locally produced. Enrichment with micronutrients was achieved by the inclusion of powdered eggshells, a source of bioavailable calcium that also contains an insulin-like growth factor (IGF-1) ^12^ reported to promote fetal and infant linear growth ^13^, and chicken liver, a readily available and affordable local animal-source food and rich source of protein, iron, zinc, vitamin A, niacin, and folate ^14, 15^. Together, the powdered eggshells and chicken liver provide a rich source of growth-promoting micronutrients with the potential to overcome the micronutrient shortfalls identified in the diets of both mothers and infants in the Sumedang district ^7, 8^. To address the possible risk of exposure of these resource-poor infants to faecal contamination from all sources, including drinking water and the subsequent development of EED, all trial participants will be supplied with educational materials focused on safe water, sanitation, and food safety ^16, 17^. The overall goal of this trial is to assess the effectiveness of daily consumption of locally produced MEC by mothers during pregnancy and for five months postpartum on infant birth length and linear growth of breastfed infants to 5 months of age, among other secondary outcomes.

### Study objectives

The primary objective is to assess the effectiveness of daily consumption of MEC by mothers at two-time intervals: (i) from the 8–14 weeks of pregnancy to delivery (i.e., 28–34 weeks of consumption of MEC) on birth length, and (ii) from the 8–14 weeks of pregnancy to 5 months post-partum on attained linear growth and linear growth velocity of breast-fed infants. Secondary outcomes will include: (a) birth weight; (b) maternal haemoglobin at 35–36 weeks gestation; (c) breastmilk volume and micronutrient concentrations in breastmilk at 5 months post-partum; (d) maternal dietary intakes at 8–14 weeks and 35–36 weeks of pregnancy; and 2 and 5 months post-partum; (e) maternal micronutrient status at 35–36 weeks and 5 months post-partum; (f) micronutrient status of breastfeeding infants at aged 5 months; (g) assessment infant neurodevelopment at 2 and 5 months; and (h) incidence of morbidity at mother and infant.

### Study design and site

This study is a double-blind placebo-controlled cluster randomised trial comparing the effect of daily consumption of locally produced MEC (intervention group) compared to placebo crackers (control group) by mothers at two-time intervals: 1) from the 8–14 weeks of pregnancy to delivery; and 2) continued for up to 5 months postpartum.

The trial will be conducted in Sumedang district, which is located 50 km from Bandung City (capital of West Java) and has a population of 1.1 million and an area of ~152 square km. The climate is tropical with rainfall during most months, although often heavier from October to February with a short dry season, generally from March to September. Approximately 22% of the area is used for paddy plantations.

**Figure 1.**
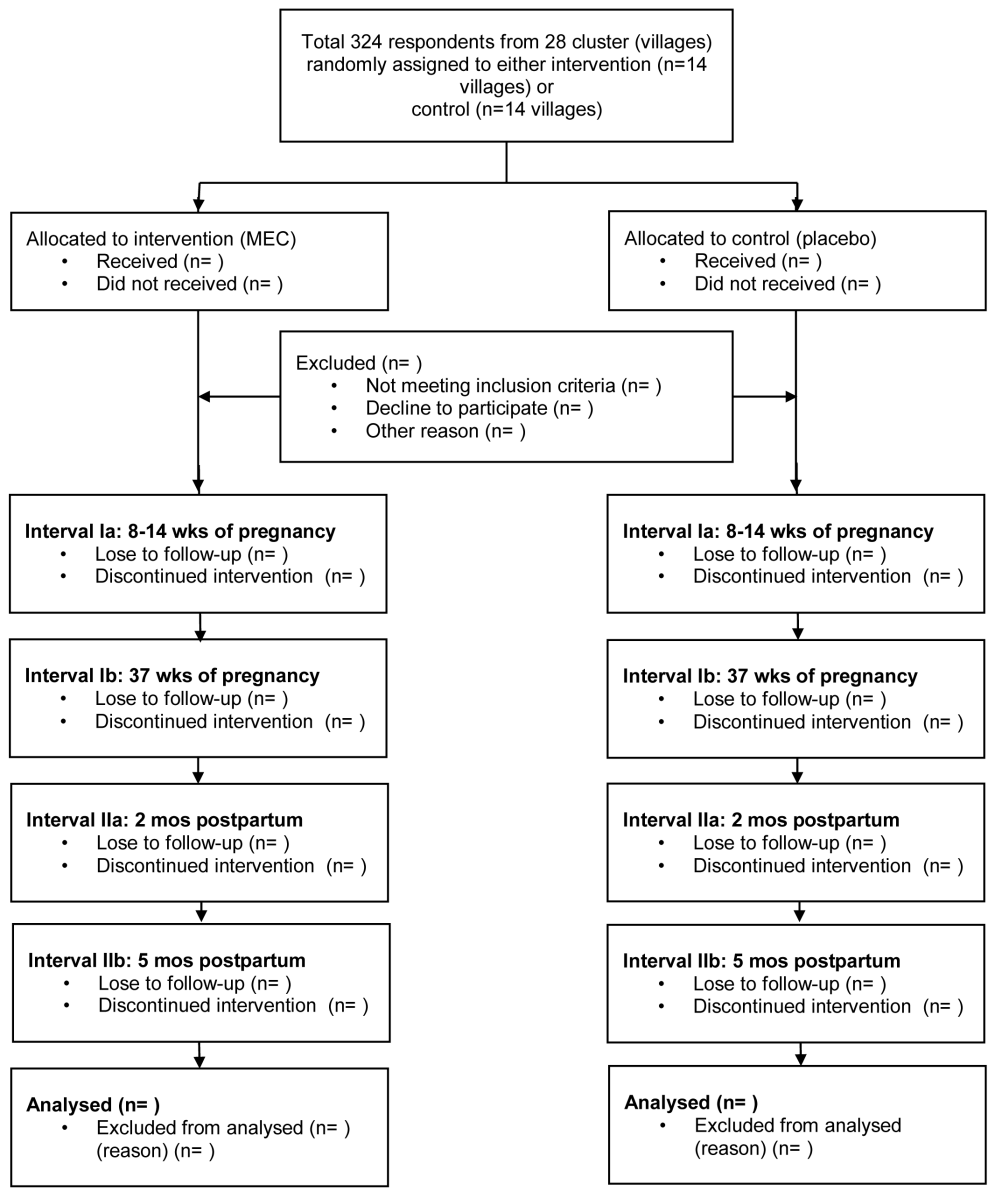
Study designs. MEC: Micronutrient-enriched Crackers.

The trial design is outlined in Figure 1. Participation in the trial is voluntary. All eligible participants will be provided with the ‘Participant Information Sheet’ that covers full details about the trial. Written informed consent will be secured from all eligible participants, who will be free to withdraw from the trial at any time.

### Randomisation, eligibility criteria, interventions, blinding, and adherence.

***Randomisation***. A total of 28 clusters (villages) located in 3 sub-districts in Sumedang district, West Java, Indonesia, will be randomly assigned to either intervention (n=14 villages) or control (n=14 villages) using a randomised sequence created by Stata 16.1 (StataCorp, College Station, TX) statistical software, stratified by villages with <100 and ≥100 infants using block sizes of 2 and 4. Cluster randomisation will be used to avoid possible bias caused by mothers in the same villages sharing MEC/placebo with each other.

***Eligibility criteria***. The research team will explain the purpose of the trial to the representatives of the midwives in the community health centres ( *Puskesmas*) in the study area and cadres (community health workers) in each participating village. Midwives will identify eligible pregnant women through the pregnancy register. Inclusion criteria are 1) pregnant women aged 19–35 years old; 2) gestational age 7–13 weeks at the time of screening (based on the last reported menstrual period); 3) willing to take part in an intervention study at 8–14 weeks gestation, and 4) permanent residents who do not plan to move in the next year. Exclusion criteria include mothers who have: 1) chronic diseases such as hypertension, diabetes, high blood cholesterol, high blood uric acid, hypercholesterolemia, a history of tuberculosis, and other chronic diseases that require prolonged treatment, such as cancer and heart disease, or epilepsy at enrolment; 2) history of preeclampsia/eclampsia and gestational diabetes in their previous pregnancy; 3) risk of chronic energy deficiency (mid-upper arm circumference < 23.5 cm); 4) severe anaemia (Hb cutoff. <7 g/dL); and 5) history of allergy to the chicken liver and/or eggs. Pregnancy will be re-confirmed by a rapid urinary test after gaining written informed consent.

The drop-out criteria include 1) preeclampsia, eclampsia; 2) dead infant/stillbirth; 3) twin delivery; and 4) infants born with congenital anomalies. Low birth weight (LBW) infants, premature infants, and infants who are not breastfed will be excluded from the assessment of both breastmilk volume and exclusive breastfeeding status by the deuterium oxide dose-to-mother technique. However, their data on growth and development will be collected.

***Interventions***. Eligible pregnant women from these villages who have agreed to participate will receive daily interventions comprising 75 grams of locally produced MEC or placebo crackers.

*Micronutrient-enriched crackers* (MEC)

The daily dose of MEC to be consumed is calculated based on the mother's nutritional requirements and the micronutrient content of the MEC, as shown in Table 1. The target consumption of crackers (i.e., 75 grams /day) is designed to meet the minimum mean prevalence of adequacy (MPA) for eight nutrients (i.e., at least 90%, except folate and B12). MPA for each micronutrient was estimated using the Estimated Average Requirement (EAR) cut-point method, as reported earlier ^8^.

**Table 1.** The basis for calculating the daily dose of MEC to be consumed (i.e., 75 g package).

Micronutrient	EAR	Actual intake ^19^			MEC per package (75 gram) *
		Median	IQR	PA, %	
Vitamin A (µg RAE)	450	827	(607-1165)	92	314 **
Thiamine (mg) ^a^	1.2	3.4	(2.7-4.3)	99	0.3
Riboflavin (mg) ^a^	1.3	4.4	(3.5-5.6)	100	0.4
Niacin (mg) ^a b^	13	19.8	(16.5-22.4)	95	3.7
Vitamin B6 (mg) ^a^	1.7	2.7	(2.4-3.1)	99	0.2
Folate (µg)	450	546	(421-698)	68	74
Vitamin B12 (µg)	2.4	3.4	(2.6-4.4)	79	2.3
Calcium (mg) ^c^	800	1286	(1168-1517)	99	556
Iron (mg) ^d^	11.7	30.1	(23.8-37.7)	99	3.6
Zinc (mg) ^e^	7	18.8	(16.6-21.5)	100	4.4
MPA				93%	

EAR: Estimated average requirement; IQR: Interquartile range; PA: Prevalence of adequacy; MEC: Micronutrient-enriched cracker; MPA: Mean Prevalence of Adequacy All values for EARs are from WHO/FAO ^20^ unless otherwise stated a Back calculated from Recommended Nutrient Intake (RNI) ^20^ b Does not include niacin from tryptophan. c EAR from IOM ^21^ d EAR from IOM ^22^ assuming 10% bioavailability e EAR from IZiNCG ^23^ assuming bioavailability from a mixed or refined vegetarian diet *Taking into account the retention values after processing **Tolerable Upper Intake Level (U.L.) for adults: 3000 μg RAE/day of preformed vitamin A ^22^ Note: 1.   The content of vitamin A, calcium, iron, and zinc was measured using the MEC chemical analysis. Meanwhile, the content of thiamine, riboflavin, niacin, vitamin B6, folate, and vitamin B12 were measured by using the Food Composition Table (FCT). 2.   EAR used in the table is the EAR of lactating women because it is higher if compared to the EAR of pregnant women, so that it may cover EAR of both lactating women and pregnant women

The MEC is prepared from chicken liver and powdered chicken eggshells [a source of bioavailable calcium and IGF-1 plus additional ingredients: tapioca flour, wheat flour, eggs, salt, mushroom bouillon, margarine, and seasoning. The chicken liver is cleaned following the Food and Drug Supervisory Agency ( *BPOM*) 2016 number 21 ^18^. MEC products have been analysed in *Saraswanti Indo Genetech* (SIG) Laboratory, accredited by KAN LP-184-IDN with SNI ISO/IEC 17025: 2008I ( Table 2).

**Table 2.** Micronutrient-enriched cracker (MEC) and placebo nutritional content and contamination assessment (per 75 g).

Parameter	MEC	Placebo
**Macronutrient**		
Protein (%) ^a^	13.0	6.1
Fat (%) ^a^	40.4	40.1
Carbohydrate (%) ^a^	39.6	48.5
Cholesterol (mg) ^a^	108.8	14.2
Total energy (kcal) ^a^	430.1	434.2
**Micronutrient**		
Vitamin A (µg RAE) ^b^	314 **	13.3
Thiamine (mg) ^b^	0.3	0.2
Riboflavin (mg) ^b^	0.4	0.2
Niacin (mg) ^b^	3.7	2.4
Vitamin B6 (mg) ^b^	0.2	0.1
Folate (µg) ^b^	74.5	47.1
Vitamin B12 (µg) ^b^	2.3	0.1
Calcium (mg) ^a^	553.9	185.1
Iron (mg) ^a^	3.6	3.2
Zinc (mg) ^a^	4.4	3.9
Sodium (mg) ^a^	333	404
**Chemical and biological contamination analysis**		
Pb	Not detected	Not detected
Hg	Not detected	Not detected
Cd	Not detected	Not detected
As	Not detected	Not detected
Sn	Not detected	Not detected
*Salmonella sp.*	Negative	Negative
*Bacillus cereus*	<10	<10
*Enterobacteriaceae*	<10	<10
*Coagulase positive* *staphylococci*	<10	<10

^a^Laboratory analysis; ^b^Calculated based on recipe *Taking into account the retention values after processing **Tolerable Upper Intake Level (UL) for adults: 3000 μg RAE/day of preformed vitamin A

*Placebo crackers*

Wheat flour is the primary ingredient, with *Pangium edule* seeds added to mimic the colour of MEC, plus the same additional ingredients used in the preparation of the MEC, excluding the liver and eggshells. Analysis of the placebo crackers is also shown in Table 2.

Products (MEC and placebo) will be packed in food-grade aluminium foil and stored at room temperature. The product’s shelf-life is one year and was tested by the Food Technology Laboratory of *Universitas Pasundan.* Expiry dates will be checked by research assistants before household distribution of the products.

***Blinding***. The MEC and placebo will be identical in size, colour, and packaging to ensure robust allocation concealment. MEC or placebo packages will be coded (A/1 or B/2), and only the production manager will know the allocated codes for the MEC or placebo packages. All the research assistants, laboratory personnel, and statistician will remain blinded to the intervention. The production manager will be instructed not to share the code with any of the investigators involved in the trial until the primary outcomes have been analysed statistically or as requested by the Ethics Committee and/or Data Safety Monitoring Board (DSMB).

***Adherence***. Participants will be asked to save the product packaging and any remaining products, which will be collected weekly for weighing by a field assistant. To increase adherence, all respondents will be offered the seasoning option of spicy flavours; these seasonings will be given by request. At the beginning of the trial, mothers will be instructed not to share their crackers with other family members. However, to avoid this practice, we will provide mothers with a family package (i.e., 150 gr of MEC or placebo) at the beginning of the study; and at weekly intervals during the study, on request.

### Measurements

Details of the measurements, micronutrient biomarkers assays, and the data collection schedule are given in Table 2. Data collection will be carried out by interviews, observations, examinations, and measurements, using trained, experienced research assistants and, where necessary, instruments that have been calibrated, pre-tested, and standardised. All biological samples (i.e. blood, breastmilk, faeces, urine, and saliva) will be collected and transported, maintaining a cold chain.

*Socio-demographic and health data*. This will include pregnancy history and the mother's pre-pregnancy height and weight, obtained through interviews by trained research assistants at 7–13 weeks of gestation.

*Anthropometric measurements*. Maternal height and weight and infant length and weight will be measured by trained research assistants using standardised techniques and calibrated equipment ^24^. Infant birth weight to the nearest 100 g will be measured by trained research assistants in the first 24 hours after birth using a digital scale (SECA 334, Seca GmbH & Co. KG., Hamburg, Germany) calibrated weekly. Birth length will be measured to the nearest 1 mm within 24 hours after birth by a research assistant trained in the WHO protocol for recumbent length ^18^ using portable infantometers (SECA 417, Seca GmbH & Co. KG., Hamburg, Germany). Both inter-and intra-examiner technical error of the measurement (TEM) for each anthropometric measurement will be calculated based on 20 infants. Accuracy is analysed by observing the tendency of mean results measured by research assistants compared with an experienced anthropometrist and then testing for statistical significance. Any significant differences observed will be evaluated and research assistant will be retrained. Measurements will be done twice, and third measurement will be done if the difference between the 1 ^st^ and 2 ^nd^ measurement exceeds the maximum allowable difference (0.1 kg for weight, 0.7 cm for length or height, 0.5 cm for circumference) ^25^. A detailed timeframe of proposed data collection is summarised in Table 2.

*Blood sample collection and analysis for micronutrient biomarkers*. Trained phlebotomists will draw non-fasting venipuncture blood samples from all willing mothers and infants into EDTA and trace-element-free evacuated tubes (BD Vacutainer, BD, Oxford, UK), kept at -4°C in cool boxes for transfer to the laboratory within 4–5 hours after collection, and refrigerated immediately prior to centrifugation. The time of blood collection, the time elapsed since the last meal, and presence of symptoms of infection will be recorded. Blood will be centrifuged (10 min at 2500 × g, 23°C), serum separated and aliquoted using trace-element free techniques, and then frozen on the day of collection for storage at −20°C until analysis.

Haemoglobin will be assayed on an EDTA blood sample from a complete blood count based on an automated haematology analyser (Sysmex XP-100, Sysmex Corporation, Kobe, Japan). Serum ferritin, soluble transferrin receptor (sTfR), retinol-binding protein (RBP), and two inflammatory biomarkers (C-reactive protein (CRP) and α-1-acid glycoprotein (AGP)) will be analysed by a combined sandwich ELISA method ^26^, and serum zinc and selenium by inductively coupled plasma mass spectrometry (ICP-MS). An external control (UTAK, Utak Laboratories Inc., Valencia, USA) will be used to validate the accuracy of the assays, while precision checks will be performed on matrix-matched pooled samples.

*Human milk volume* (mL/d). The volume of milk consumed by the infant over a 14-day period will be derived from data generated by the deuterium oxide dose to mother (DTM) technique over 14 days. Predose saliva samples will be collected on day 0 from both mother and infant, after which mothers will be given an accurately measured oral dose of diluted deuterium oxide as described earlier ^4^. Next, a 3-postdose saliva collection design will be used whereby samples will be collected from the mother and infant on days 2 or 3; 8 or 9; and days 14 as validated and described by Liu *et al.* ^27^. Care will be taken to ensure that neither mother nor infant had consumed food or fluid at least 30 min or 15 min, respectively, prior to each saliva collection. All saliva samples will be frozen on the day of collection and stored at −20°C until analysis of deuterium enrichment by Fourier transform infrared spectrometry (Agilent 4500, Agilent Technologies, Dansbury, USA) ^28^. A fully Bayesian framework using a gradient-based Markov chain Monte Carlo approach ^27^ will be used to calculate the average amount of human milk intake over a 14-d period and to distinguish exclusive breastfed infants from non-exclusive breastfed infants.

*Breastmilk collection and micronutrient analysis*. A full breast expression of human milk samples from one breast will be collected in the morning on day 14 (after deuterium oxide dosing) at 5 months postpartum using a breast pump (Medela Harmony Manual Breastpump, Medela AG, Baar Switzerland), avoiding all sources of adventitious contamination. After transfer into an acid-washed ^29^ trace element–free plastic bottle and gentle mixing, aliquots (1 mL) of whole human milk will be transferred into acid-washed microtubes. The latter will be covered in aluminium foil to minimise degradation of photosensitive vitamins, frozen on the day of collection, and stored at −80°C until analysis.

Human milk retinol and vitamin E concentrations will be expressed per g fat in addition to unit volume ^4^ based on milk fat concentrations measured via the Creamatocrit method ^30^. Mineral analysis (sodium, magnesium, phosphorus, potassium, calcium, iron, copper, zinc, and selenium) in human milk will be carried out in the Centre for Trace Element Analysis, Department of Chemistry, the University of Otago by ICP-MS (Agilent 7900, Agilent Technologies, Santa Clara, USA), as previously described ^4^. Precision and accuracy of the minerals will be checked against in-house pooled samples and a multielement reference standard (SRM 1846, infant formula) from the National Institute of Standards and Technology, Gaithersburg, USA ^31^.

Analysis of the vitamins will be conducted at the USDA Agricultural Research Service Western Human Nutrition Research Center (WHNRC). Free thiamin, thiamin monophosphate (TMP), and thiamin pyrophosphate (TPP) will be analysed by HPLC with fluorescence detection ^32^. Riboflavin, flavin adenine dinucleotide (FAD), flavin mononucleotide (FMN), nicotinamide, nicotinic acid, nicotinamide adenine dinucleotide (NAD), nicotinamide mononucleotide (NMN), nicotinamide riboside (NR), pantothenic acid, pyridoxal (PL), pyridoxine (PN), pyridoxamine (PM), pyridoxal-5-phosphate (PLP), biotin, and tryptophan will be analysed in multiple reaction monitoring mode by ultra-performance liquid chromatography (UPLC) MS/MS ^33^ with a SCIEX Exion-LC AD UHPLC coupled to a SCIEX 6500+ QTRAP mass spectrometer (SCIEX, Redwood City, CA, USA). Vitamin B-12 (cobalamin) will be analysed by competitive chemiluminescent enzyme immunoassay (IMMULITE 1000; Siemens, Washington DC, USA) as previously described ^32^. Preformed retinol and three provitamins A carotenoids (α-carotene, β-carotene, and β-cryptoxanthin), α-tocopherol, and γ -tocopherol will be determined by an Agilent 1260 HPLC system with multiwavelength detection ^34^ (Agilent Technologies, Santa Clara, USA). Vitamin E will be expressed as tocopherol equivalents (TE), defined as α-tocopherol + 0.25 γ-tocopherol ^35^. Total vitamin concentrations derived from different vitamers will be estimated by calculating the amount of the free or main form of the vitamin within each vitamer based on molecular weights (e.g. the amount of riboflavin in FAD for vitamin B2). The sum of these amounts will be used as total vitamin concentration.

***Dietary intakes of the mother***. Data will be collected for three non-consecutive days by 24-hour self-reported weighed food records (WFR) as previously reported ^5, 36^; using calibrated digital scales with a precision of 2 g (Kitchen Scale EK3650, Camry Electronic Ltd., Guangdong, China). The mothers will perform the WFRs following a training and practice session. Mothers will also receive video tutorials and leaflets to help them with the weighing techniques. The day after the weighing schedule, a research assistant will call the mother and examine photographs of the WFR forms which have been filled by the mother to check on the weights recorded. If the research assistant finds some unreasonable data on the WFR forms, the research assistant will show the food photographs book to the mother to confirm the size of the food consumed by the mother. Maternal nutrient intakes will be calculated using a locally produced Indonesian food composition table that incorporates the mandatory micronutrient fortification of all wheat flour products ^37^.

*Neurodevelopmental status of infants*. Infant neurodevelopment will be measured at 2 and 5 months using the Ages and Stages Questionnaires, third edition (ASQ-3), which has been translated into Bahasa Indonesia and validated ^38, 39^. Neurodevelopmental assessments will be conducted by the trained research assistant.

***Maternal and infant morbidity calendars***. Participants will be trained to record any episodes of illness that occur over 30 days during selected periods ( Table 3) during the trial using the morbidity calendar provided. The monthly infant morbidity calendars will be recorded by the mothers.

**Table 3.** Schedule of enrolment, interventions, and assessments. 1 ^st^ phase in pregnancy, at birth, and 2 ^nd^ phase in lactation period.

Outcome	1 ^st^ phase		At birth	2 ^nd^ phase		Assessment method
	7-14w	35-36w		2m	5m	
**ENROLMENT**						
Data on pregnant women	X					Data collection from village midwives
Eligibility screen	X					Assessment is based on screening and inclusion and exclusion criteria.
**Informed consent**	X					Explanation to respondents individually
Allocation	X					
**INTERVENTION**						
MEC consumption	X	X		X	X	
Placebo consumption	X	X		X	X	
**ASSESSMENT**						
MEC/Placebo consumption compliance	X	X		X	X	Weighing the MEC/Placebo consumed daily.
Sociodemographic	X					Standardised pre-tested questionnaire
Pregnancy history	X					Standardised pre-tested questionnaire
Maternal morbidity (monthly)	X	X		X	X	Self-reported by respondents
Infant morbidity (monthly)				X	X	Self-reported by respondents
Iron supplement tablets/supplement consumption of mother	X	X		X	X	Self reported by respondents, recorded in compliance form
MEC/Placebo distribution form	X	X		X	X	Reported by the production manager
Food intake and immunisation of infant				X	X	Standardised pre-tested questionnaire
Hygiene and sanitation	X	X		X	X	Standardised pre-tested questionnaire and direct observation
Mother's blood pressure	X	X		X	X	Measurements with Automatic Blood Pressure Monitor HEM-7120, Omron Healthcare, Tokyo, Japan.
Mother's mid-upper arm circumference	X					Measurement with a measuring tape (SECA 212, Seca GmbH & Co. KG., Hamburg, Germany)
Mother's self-reported weight before pregnant	X					
Mother's height	X				X	Measurement by stadiometer (SECA 213, Seca GmbH & Co. KG., Hamburg, Germany)
Mother's weight	X	X		X	X	BIA method measurement by electronic scale (TANITA SC-240 MA, Tanita Corporation, Tokyo, Japan)
Increase in maternal weight during pregnancy	X	X				Mother’s self-reported weight before pregnant and maternal weight measurements with an electronic scale (TANITA SC-240 MA, Tanita Corporation, Tokyo, Japan)
Mother's body composition	X	X		X	X	BIA method measurement by electronic scale (TANITA SC-240 MA, Tanita Corporation, Tokyo, Japan)
Infant head circumference (at birth: within 24 hours)			X	X	X	Measurement with a measuring tape (SECA 212, Seca GmbH & Co. KG., Hamburg, Germany)
Infant's mid-upper arm circumference (at birth: within 24 hours)			X	X	X	Measurement with a measuring tape (SECA 212, Seca GmbH & Co. KG., Hamburg, Germany)
Infant's length (at birth: within 24 hours)			X	X	X	Measurement with an infantometer (SECA 417, Seca GmbH & Co. KG., Hamburg, Germany)
Infant's weight (at birth: within 24 hours)			X	X	X	Measurements with infant scales (SECA 334, Seca GmbH & Co. KG., Hamburg, Germany)
Infant's nutritional status			X	X	X	Based on WHO Anthro z-scores
Infant's development				X	X	Ages and Stages Questionnaire (ASQ-3)
Mother's food intake	X	X		X	X	3-day non-consecutive self-reported weighed food record using a food scale (Camry EK3650, Camry Electronic, Guangdong, China) in combination with a food recall
Breastmilk intake/volume					X	Calculated with Deuterium Oxide Dose to Mother Technique
Breastmilk samples from 1 full breast					X	Breastmilk is collected with a Medela Harmony Manual Breastpump, Medela AG, Baar Switzerland.
Mother and infant saliva					X	Deuterium oxide levels will be analysed using FTIR (Agilent 4500, Agilent Technologies, Dansbury, USA)
Screening of maternal hemoglobin (Hb)	X					Blood drawing from fingertips - HemoCue 201+, HemoCue AB, Angelholm, Sweden
Screening of random blood sugar	X					Blood drawn from the fingertips - Accutrend Plus, Roche Diagnostic GmbH, Mannheim, Germany
Mother's blood uric acid	X	X			X	Blood drawing from fingertips – FamilyDr Uric Acid Meter, General Life Biotechnology Co. Ltd., Taipei, Taiwan.
Mother's total cholesterol	X	X			X	Blood drawn from the fingertips - Accutrend Plus, Roche Diagnostic GmbH, Mannheim, Germany
Mother’s Complete Blood Count (CBC)		X			X	Sysmex XP-100, Sysmex Corporation, Kobe, Japan
Infant's Complete Blood Count (CBC)					X	Sysmex XP-100, Sysmex Corporation, Kobe, Japan
Mother’s CRP, AGP, Ferritin, sTfR, RBP		X			X	Analysis at Juergen Erhardt's Laboratory, VitMin Lab, Willstaett, Germany (combined sandwich ELISA method)
Infant's CRP, AGP, Ferritin, sTfR, RBP					X	Analysis at Juergen Erhardt's Laboratory, VitMin Lab, Willstaett, Germany (combined sandwich ELISA method)
Mother's zinc and selenium serum		X			X	Analysis at the Center for Trace Element Analysis Laboratory, University of Otago, New Zealand (ICP-MS) (Agilent 7900, Agilent Technologies, Santa Clara, CA, USA)
Infant's zinc and selenium serum					X	Analysis at the Center for Trace Element Analysis Laboratory, University of Otago, New Zealand (ICP-MS) (Agilent 7900, Agilent Technologies, Santa Clara, USA)
Major elements in breastmilk (Na, Mg, P, K, Ca) and trace elements (Fe, Cu, Zn, Se)					X	Analysis at the Center for Trace Element Analysis Laboratory, University of Otago, New Zealand (ICP-MS) (Agilent 7900, Agilent Technologies, Santa Clara, USA)
Breastmilk analysis (free thiamine, TMP, TPP)					X	Analysis at the USDA, ARS Western Human Nutrition Research Center, Davis, CA, USA (HPLC-FLD) (Agilent 1200, Agilent Technologies, Santa Clara, USA)
Breastmilk analysis (cobalamin)					X	Analysis at the USDA, ARS Western Human Nutrition Research Center, Davis, USA (Competitive Chemiluminescent Enzyme Immunase) (IMMULITE 1000; Siemens, Washington DC, USA)
Breastmilk analysis (riboflavin, flavin adenine dinucleotide (FAD), flavin mononucleotide (FMN), nicotinamide, nicotinamide adenine dinucleotide (NAD), pantothenic acid, pyridoxal (P.L.), pyridoxine (P.N.), and biotin)					X	Analysis at USDA, ARS Western Human Nutrition Research Center, Davis, CA, USA (ultra-performance liquid-chromatography tandem mass spectrometry with a SCIEX EXionLC AD UHPLC coupled to a SCIE 6500+ QTRAP mass spectrometer)
Breastmilk analysis (preformed retinol and three provitamins A carotenoids (α-carotene, β-carotene, and β-cryptoxanthin), α-tocopherol, and γ-tocopherol)					X	Analysis at the USDA, ARS Western Human Nutrition Research Center, Davis, USA (HPLC) (Agilent 1260, Agilent Technologies, Santa Clara, USA)
Daily-use water and drinking water checks					X	Analysis in the University of Padjadjaran Laboratory (Membrane Filter Technique)
Urinary iodine					X	Analysis in Indonesian laboratory
*Enteropathy* and microbiome from infant feces				X	X	Samples are stored frozen for further examination (DNA sequencing, microbiome analysis, EED)

*Health and nutrition education training*. All m **i**dwives in trial villages will receive training by a gynaecologist on applying the delayed cord clamping method during the birth of all neonates. The method is recommended by WHO to increase iron reserves in term and preterm infants who do not need immediate resuscitation ^40^. All trial participants will receive nutrition education provided by the research team on the following topics: (i) the first 1000 days of life; (ii) balanced-nutrition during pregnancy and lactation (including raising awareness to limit high intake of purines and cholesterol); (iii) water, sanitation, and hygiene (WASH) including five key messages for making food consumed safer: washing hands and washing utensils for eating and cooking, separating raw and cooked ingredients, cooking thoroughly (boiling water, cooking all types of food), keeping food at safe temperatures, and using clean water and safe materials; (iv) and good breastfeeding practices ^16, 17^.

### Ethical approval and registration

This trial conforms to the principles outlined in the Declaration of Helsinki 2013 ^41^, and has been approved by Indonesian Health Research Ethics Committee, National Institute of Health Research and Development (HREC-NIHRD), reference number LB.02.01/2/KE.496/2020 and LB.02.01/2/KE.503/2021; protocol version 1.4 (3 ^rd^ August 2021). Any important protocol modifications will be discussed with the Data Safety and Monitoring Board (DSMB) and then with the ethics committee to establish whether any amendments are needed. The protocol was registered on the U.S. National Library of Medicine (ClinicalTrials.gov) with the identifier NCT04564222 on 25 ^th^ September 2020 ( https://clinicaltrials.gov/ct2/show/NCT04564222).

### Safety considerations including intake of potential contaminants and cholesterol

*Contaminants.* Analysis of both intervention and placebo products, performed by SIG laboratory, revealed both products are within the ‘safe limits’ for microbes ^42^ and heavy metals ^43^, as shown in Table 1.

*Cholesterol.* Cholesterol typically increases during pregnancy, especially during the second and third trimesters, when it may exceed normal physiological levels, and increase the risk of complications during pregnancy (i.e., preeclampsia, premature birth, and gestational diabetes) and impair the vasodilator function of placental blood vessels ^44^. A daily dose of MEC cholesterol concentration is 107.2 mg/day, which falls within the safe limit of daily dietary cholesterol intake (i.e., < 300 mg/day) ^44^. The serum cholesterol levels of the trial participants will be monitored before the trial begins, repeated at 35–36 weeks' gestation, and at endline. Respondents with hypercholesterolemia (cholesterol serum >240 mg/dL) at enrolment will not be included, and referred to the nearest healthcare provider for further treatment.

Therefore, in light of the above discussion and the precautionary strategies implemented, the possibility of women developing undesirable effects from the daily consumption of the designated amount of MEC appears minimal. Any of the potential undesirable pregnancy outcomes itemised above will be recorded by the midwives, with confirmation from the Clinical Monitor, and subsequently reported to the DSMB.

### Sample size calculation, data management, and statistical analysis

All parameters used in the sample size calculations are reported in Table 4. The design effect, due to clusters, has been estimated using previous data collected in the area. Standard operating procedures have been prepared with respect to all measurement procedures, trial management, quality assurance, data management, and statistics and will be followed by the relevant research assistants to ensure standardisation of all processes.

**Table 4.** Parameters used in sample size calculation for secondary outcomes.

Measure	Observed mean	Observed SD	Difference to detect	ICC	Design effect *	Sample size **
Birth length (cm) ^a^	49.70	2.20	1.0	0.010	1.10	91
Infant length at 5 months (cm) ^b^	62.98	2.20	1.0	0.010	1.10	91
Birth weight (g) ^a^	3013	514	200	0.010	1.03	118
Haemoglobin at 3rd trimester (g/dL) ^c^	12.58	1.18	0.5	0.048	1.48	138
Volume of breastmilk at 5 months post- partum (mL/day) ^d^	787	148	70	0.010	1.10	84
Micronutrient concentrations of breastmilk at 5 months post-partum (Vit A, μg/L) ^d^	634	281	130	0.036	1.36	107
Maternal diet at 5 months post-partum (Vit A, μg RAE/d) ^e^	478	244	110	0.010	1.10	93
Maternal micronutrient status at 5 months post-partum (Ferritin, µg/L) ^a^	35.79	29.70	13.0	0.010	1.10	98
Infant micronutrient status at aged 5 months (Ferritin, µg/L) ^a^	46.65	35.58	15.0	0.010	1.10	106

ICC: intra-class correlation *Design effect: based on average size of cluster = 11 **Required sample size per group ^a^Unpublished data; ^b^Data from observation by Diana *et al.* ^7^; ^c^Data from observation by Diana *et al.* ^3^; ^d^Data from observation by Daniels *et al.* ^4^; ^e^Data from observation by Rahmannia *et al.* ^8^

*Sample size calculation.* For time interval 1 (i.e., from the second trimester of pregnancy (8–14 weeks) to delivery), a minimum sample size of 182 is required to detect a 1 cm difference in birth length between intervention and control (this is approximately 0.5 SD from WHO Growth Chart ^45^ with a power of 0.90, a statistical significance of 0.05, and a design effect of 1.1 due to village clusters. For time interval 2 (i.e., from the second trimester of pregnancy to 5 months old), based on an average growth velocity of 3.0-3.2 cm/month ^45^, we propose to detect a 0.7 cm difference in growth velocity between intervention and control. With a power of 0.90, a statistical significance of 0.05, assuming a standard deviation of 2.2 cm/month, a within-person correlation of 0.73, and a design effect of 1.1 due to village clusters, a sample size of 204 would be required (102 in each group). With exclusion due to stillborn or premature birth (~10%, i.e., n~15 in each group), or failure to breastfeed (~15%, i.e., n~23 in each group), drop-out/loss to follow-up of approximately 25% (i.e., n~39 in each group), the final sample size is ~ 324. This sample size will also allow the detection of differences in secondary outcome measures, as shown in Table 4.

*Data management*. Experienced research assistants specially assigned for this task will check the accuracy and completeness of data collected. All data will be doubly entered and checked for identical entries using RedCap software. Any errors will be flagged and re-checked with hard copy data. The data manager will conduct data cleaning to ensure values are within the acceptable/plausible ranges and identify missing values. All identifiers and personal information of respondents will be coded prior to data analysis and storage. Datasets will be de-identified in accordance with the Safe Harbor method before sharing.

*Statistical analysis*. Primary analyses will be undertaken as intention to treat. The mean number of crackers consumed overall and compliance rates will be examined. Only live-born infants will be included in all analyses. Intervention groups will be compared using mixed-effect regression models using cluster (village) as a random effect. The primary outcomes for time interval 1 are the birth length, and for time interval 2 are both attained and incremental infant linear growth at five months postpartum. Analyses for time interval 2 will be conducted with adjustment for weight-for-age and breastfeeding practice (exclusively/partially breastfeeding), where appropriate. Model assumptions will be checked using standard procedures, and variables log-transformed when necessary. Guidelines for reporting will be followed as outlined in the Consolidated Standards of Reporting Trials (CONSORT) statement: extension to cluster randomised trials ^46^. After the trial is complete, the full protocol and unidentifiable dataset can be accessed by the public via the corresponding author.

***Safety monitoring.*** The Clinical Monitor will conduct an ongoing monitor on of any adverse events, which will be recorded on the adverse events forms. All safety data will be reviewed annually by the Data Safety Monitoring Board (DSMB), which includes an independent group of experts charged with reviewing research data for data quality and integrity, adherence to the protocol, participant safety, research conduct and progress, and decisions regarding the continuation and/or modification suspension/termination of the research project. The DSMB is independent of any professional or financial conflict of interest (COI) with the research project and/or research investigators.

Given the short period of data collection and the anticipation that no highly potential harm/risk will be expected, the trial will continue until the end of data collection without interim data analysis. The research team will report any undesirable events to the DSMB and the Ethics Committee. The DSMB may call for an interim analysis if it is required. The clinical trial termination will be carried out based on the DSMB and/or Ethics Committee's recommendation.

### Withdrawal of participants

In cases when a participant withdraws from the trial, biological samples of the withdrawn participant will not be analysed, unless consent is given. However, any information generated from the samples up to the time of withdrawal will be used. The Clinical Monitor may also ask for tests for the participant’s safety. Reasons for any withdrawals will be sought, and participant follow-up will be carried out regarding any unresolved adverse events.

### Health protocols for COVID-19 prevention

For the prevention of COVID-19 during the research data collection process, standardised operational procedures will be followed. The entire research team, trial participants, involved in data collection activities, and the production staff producing the MEC/placebo must all comply with the Decree of the Minister of Health of the Republic of Indonesia ^47^ or any updated regulations. The research team, cadres, and trial participants will be screened for risk of COVID-19 before every visit using questionnaires. High-risk respondents will be rescheduled after an appropriate time interval to reduce risk or when they return a negative COVID-19 test. All research assistant personnel are responsible for collecting the data in the field using standard Personal Protective Equipment (PPE). Contact tracing procedures will be followed if member(s) of the research team are positive for COVID-19. Home self-isolation will be carried out by those who have close contacts, are suspected, or have mild positive symptoms after being swabbed for PCR/antigen test at the health service. Those who are positive for COVID-19 with moderate and severe symptoms will be referred to the nearest hospital. All research activities will be performed as quickly as possible while maintaining health protocols without compromising the quality of the data collected.

## Study status

The study has recruited 324 pregnant women (April 2022). The final mother-infant pairs will be due to be followed up in March 2023.

## Discussion

In the proposed study, we aim to overcome the shortfalls in the micronutrient content of the diets of women from the Sumedang district and alleviate both micronutrient deficiencies and sub-optimal breastmilk concentrations ^4, 5^ by augmenting their habitual diets from the 2 ^nd^ trimester of pregnancy up to the first six months of lactation with micronutrient enriched crackers (MEC), a favourite snack food in Indonesia. This will be the first study in Indonesia to use crackers based on powdered eggshells together with chicken liver, a much needed animal-source food (ASF) in the diets of mothers in the Sumedang district, in a form which is both acceptable and safe. Moreover, the MEC have a long shelf-life, an essential attribute given the many constraints linked to the consumption of ASFs identified earlier in the Sumedang district ^8^.

We aim to determine whether the daily consumption of the MEC for 6 months during pregnancy enhances the birth length, and whether by continuing their daily consumption for 5 months postpartum, both attained and infant incremental linear growth at 5 months of age can also be improved. Most RCTs in Indonesia have examined birthweight and not birth length, so whether stunting (if present) is associated with intrauterine growth retardation or develops subsequently during the early postpartum period in Indonesia is uncertain.

This study will also generate important information on the adequacy of the dietary intakes and biochemical status of micronutrients in Sumedang women and their infants at 5 months postpartum, the rate of exclusive breastfeeding using the state-of-the art DTM technique, and whether the volume and/or micronutrient composition of breastmilk is affected by consumption of MEC.

If the proposed MEC study proves effective at improving length at birth and at five months postpartum as well as improvements in other health indicators (e.g., micronutrient status and reduced morbidity), then MEC will be produced locally by women from the communities as part of a women’s community empowerment program. Participation in such a program could potentially enhance women's empowerment as our country has various voluntarily local women organisations which focus on health, nutrition, and corporate-related activities. Local women involved in MEC production will also serve as social marketing agents to ensure easy and sustained access to the MEC in the future.

Finally, on completion of the RCT, the results will be shared with participants, health staff, District Health Office representatives, and the Ministry of Health (MOH). If successful, guidelines for scaling up the program will be provided to the MOH. The guidelines will include rigorous monitoring and evaluation tools for assessing program effectiveness that is tailored to the local situation in targeted areas in Indonesia.

Furthermore, this study will generate outputs that will be a resource for the broader research community, including accurate measurements of birth length and birth weight and subsequent attained growth and velocity (length and weight) of exclusively breastfed rural Indonesian infants. Data from our control group will confirm whether stunting among these rural Indonesian infants (if present) is associated with intrauterine growth retardation, develops subsequently during the early postpartum period, or both when infants are breastfed. Such data are rarely available in Indonesia due to the lack of skills and equipment required, and time constraints, especially regarding the collection of accurate data at birth. Other data that will be generated include the rate of exclusive breastfeeding using the deuterium technique, the volume of breastmilk, micronutrient intakes and status of lactating women and infants, as well as breastmilk composition. These data will help us understand whether consumption of fortified crackers during pregnancy alone or from 8–14 weeks gestation to 5 months postpartum improves the birth length and infant linear growth, enhances breastmilk micronutrient concentrations, and whether the consumption of MEC during pregnancy and lactation warrants scaling-up in Indonesia.

## Data availability

No data is associated with this article.
