# Supplementary material for: Chicken liver and eggshell crackers as a safe and affordable animal source food for overcoming micronutrient deficits during pregnancy and lactation in Indonesia: a double-blind, randomised placebo-controlled trial (SISTIK Growth Study)
Source: Wellcome Open Res. 2022 Jun 9;7:167. [Version 1] doi: 10.12688/wellcomeopenres.17879.1 (PMC9270652; doi:10.12688/wellcomeopenres.17879.1)
Supplement: Peer review history

# Peer review: Reviewer response for version 1

**DOI:** 10.21956/wellcomeopenres.19804.r50997

**Published:** 2022-07-08

**Authors:** Chessa K. Lutter

**Affiliations:** 1 Division of Food Security and Agriculture, RTI International, Washington, DC, USA

Effective food-based interventions to prevent early childhood growth failure and micronutrient deficiencies remain elusive. In resource poor settings, newborns are at-risk of intra-uterine growth failure manifested in short stature at birth. In addition, the breast milk of poorly nourished women may be limited in some key water-soluble vitamins putting breastfed infants at risk of micronutrient deficiencies. Supplementation of women during pregnancy and during lactation may be a strategy to improve infant birth outcomes and post-natal growth trajectories and micronutrient status during the period of exclusive breastfeeding (i.e. first 6 months of life).

 In the protocol described here, the effect of a locally produced micronutrient-enriched cracker provided during pregnancy compared to controls on linear growth and linear growth velocity of breast-fed infants attained will be tested in a double-blind placebo-controlled cluster randomized trial. The trail will compare this effect at two-time intervals: a) from the 8-14 weeks of pregnancy to delivery on birth length, and (b) from the 8-14 weeks of pregnancy to 5 months post-partum. The target consumption of crackers (i.e. 75 grams/day) for pregnant women in the intervention groups is designed to meet the minimum mean prevalence of adequacy for eight nutrients (i.e. at least 90%, except folate and B12). The study will take place in Indonesia is a setting where infants and young children are at risk of stunting and micronutrient deficiencies.

 The objectives of the trial are described well and the design is appropriate for the research question. The methodology is also well thought through, and in addition to anthropometric assessment of infants and dietary assessment of mothers and infants, it includes the assessment of multiple biological samples, including, breast-milk of mothers, and the blood, faeces, urine, and saliva of infants. Assessment of infant feaces is particularly relevant given the role of gastrointestinal, including sub-clinical infections, on growth. However, unlike the in-depth presentation of the data collection and analytic methods of other biological specimens such information is lacking for the feaces and urine. This is a deficiency in the protocol that needs remedy. Also, an economic analysis of the cost of the crackers and methods of local production would be useful for assessment of sustainability after the conclusion of the trial.

 Lastly, the protocol has been registered on the U.S. National Library of Medicine (ClinicalTrials.gov) and, upon conclusion of the trial, deidentified data sets will be available upon request from the principal investigator.

Is the study design appropriate for the research question?

Yes

Is the rationale for, and objectives of, the study clearly described?

Yes

Are sufficient details of the methods provided to allow replication by others?

Yes

Are the datasets clearly presented in a useable and accessible format?

Yes

Reviewer Expertise:

Nutrition

I confirm that I have read this submission and believe that I have an appropriate level of expertise to confirm that it is of an acceptable scientific standard.

# Peer review: Reviewer response for version 1

**DOI:** 10.21956/wellcomeopenres.19804.r50998

**Published:** 2022-07-04

**Authors:** Pattanee Winichagoon

**Affiliations:** 1 Institute of Nutrition, Mahidol University, Nakhon Pathom, Thailand

This paper presents the protocol for a randomized control trial using an innovative approach by providing the locally produced micronutrient enriched (using chicken liver and egg shell) crackers (MEC) to women during pregnancy and postpartum. The primary objective is to assess the effectiveness of daily consumption of MEC by mothers from early pregnancy till 5 m postpartum on attained linear growth and linear growth velocity of breast-fed infants. The secondary objectives include maternal nutrition during pregnancy and postpartum and human milk quantity and quality on selected micronutrients, birthweight, infant’s health, micronutrient status and neurodevelopment outcomes. Overall, the study protocol was well thought out and clearly described. There are a few questions for authors to consider, as follows:

1. Since birthweight has been shown to differ by parity, parity should be included in the maternal background characteristics.
2. Since some of micronutrients in human milk may decline after the first couple months (e.g. zinc), collecting breast milk samples at 5 m may not reflect the content of these nutrients during the earlier time. While it is appreciated that the amount of additional work to collect breast milk and analyzing nutrient contents is very substantial, it will be important to at least provide the rationale for selecting this postpartum time point for the assessment of breast milk intake (quantity and quality), and how it may/may not impact on the main outcomes of the study.

Is the study design appropriate for the research question?

Yes

Is the rationale for, and objectives of, the study clearly described?

Yes

Are sufficient details of the methods provided to allow replication by others?

Yes

Are the datasets clearly presented in a useable and accessible format?

Not applicable

Reviewer Expertise:

maternal and child nutrition, including micronutrient intervention research and programs.

I confirm that I have read this submission and believe that I have an appropriate level of expertise to confirm that it is of an acceptable scientific standard.
